# Donor‐Derived Cell‐Free DNA as a Marker for the Efficacy of Daratumumab in Patients With Antibody‐Mediated Rejection Post‐Heart Transplantation: A Case Series

**DOI:** 10.1111/petr.70168

**Published:** 2025-08-24

**Authors:** Anusha Konduri, Kathryn E. Flynn, Ashley Huebschman, Bronwyn Crandall, Natalie Sinicropi, Bethany Giacobbe, Mary Zamberlan, Matthew Najor, Matthew Cusick, Heang M. Lim, Amanda D. McCormick, Kurt R. Schumacher, David M. Peng

**Affiliations:** ^1^ University of Michigan Ann Arbor Michigan USA

**Keywords:** antibody‐mediated rejection, daratumumab, donor‐derived cell‐free DNA, pediatric heart transplantation

## Abstract

**Background:**

Antibody‐mediated rejection (AMR) remains a significant complication following heart transplantation, contributing to graft dysfunction and reduced survival. Donor‐derived cell‐free DNA (dd‐cfDNA) is emerging as a non‐invasive biomarker for detecting and monitoring graft injury, correlating with episodes of rejection and response to treatment. Daratumumab, an anti‐CD38 monoclonal antibody targeting plasma cells, has shown promise in treating AMR. We present a case series of pediatric and young adult heart transplant recipients demonstrating donor‐derived cell‐free DNA's potential utility in monitoring for AMR and the effect of therapies including daratumumab.

**Case Descriptions:**

We report five cases showing that elevated dd‐cfDNA correlated with pathological AMR (pAMR), and treatment with daratumumab improved both pAMR and dd‐cfDNA levels. Most of our patients had persistently elevated donor‐specific antibody (DSA) as observed by MFI values; however, there was a reduction in DSA titer that corresponded with improvement in pAMR and dd‐cfDNA levels. Recurrent increases in dd‐cfDNA were also useful in guiding the need for repeat treatment with daratumumab. Although DSA levels often remained elevated despite histologic improvement, decreasing dd‐cfDNA levels correlated more closely with the resolution of AMR.

**Conclusion:**

In this case series of pediatric and young adult heart transplant recipients, our findings suggest that dd‐cfDNA can serve as a valuable biomarker for diagnosing AMR and treatment response, which are not often reflected by DSA MFI alone. Our dd‐cfDNA data supports the efficacy of daratumumab in treating AMR and may guide the need for ongoing treatment. Further studies are warranted to validate these findings and establish guidance for the use of daratumumab and dd‐cfDNA in this patient population.

**Abbreviations**
AMR: antibody‐mediated rejection
CD38: cluster of differentiation 38
cfDNA: cell‐free DNA
dd‐cfDNA: donor‐derived cell‐free DNA
dnDSA: de novo donor‐specific antibody
DSA: donor‐specific antibody
EMB: endomyocardial biopsy
HLA: human leukocyte antigen
HT: heart transplantation
IgG: immunoglobulin G
IV: intravenous
IVIG: intravenous immunoglobulin
kg: kilogram
MFI: mean fluorescence intensity
mg: milligram
mg/kg: milligram per kilogram
mg/mL: milligram per milliliter
mL: milliliter
NGS: next‐generation sequencing
pAMR: pathologic antibody‐mediated rejection
PCR: polymerase chain reaction
SNP: single‐nucleotide polymorphism

## 1 Introduction

Antibody‐mediated rejection (AMR) is a major complication following heart transplantation (HT) leading to graft dysfunction and decreased graft survival [1]. AMR has been shown to limit long‐term survival in HT patients and has been traditionally diagnosed and monitored with invasive endomyocardial biopsies (EMB) [1]. Donor‐derived cell‐free DNA (dd‐cfDNA) is increasingly used as a non‐invasive alternative to EMB [2, 3]. Tissue damage in the graft leads to the release of cell‐free DNA (cfDNA), which circulates throughout the body. Advancements in next‐generation sequencing (NGS) now enable the detection and measurement of fragmented DNA or specific single‐nucleotide polymorphisms (SNPs) in blood, facilitating the quantification of dd‐cfDNA. In transplant recipients, dd‐cfDNA levels are typically low during stable conditions. During rejection episodes, dd‐cfDNA may increase and decrease following therapeutic intervention, making it a promising biomarker for early detection of graft injury and monitoring of treatment response [4].

Therapies for managing AMR in HT recipients include plasmapheresis, corticosteroids, intravenous immunoglobulins (IVIG), rituximab, and bortezomib [1]. Daratumumab, a human IgG1κ monoclonal antibody targeting CD38 on plasma cells, has gained attention as a promising therapeutic option [5, 6]. Originally used as a therapy for conditions like multiple myeloma, amyloidosis, and autoimmune diseases, daratumumab is now being explored for its potential to address AMR in transplant patients, as it effectively depletes CD38‐expressing plasma cells, involved in the production of pathologic antibodies. There is growing evidence supporting the use of daratumumab in AMR management, particularly in kidney transplant recipients, with reported positive clinical outcomes [7, 8]. Recently, case reports have highlighted its use in HT patients, further expanding its potential application [5, 6].

In this case series, we describe pediatric and young adult HT recipients with AMR who received daratumumab as part of their therapy and present their correlative dd‐cfDNA as a marker of treatment response.

Manufacturer recommendations for daratumumab administration in adult patients are to dilute the first dose of daratumumab in 1000 mL and subsequent doses in 500 mL, resulting in a final concentration between 0.4 and 4.5 mg/mL. To minimize infusion volume in pediatric patients, especially those weighing less than 40 kg, our center has begun targeting final concentrations rather than total volume for administration. We found that concentrations of 1.2 mg/mL for the initial dose and 2.4 mg/mL for subsequent doses allowed for minimization of total volume administered while preventing infusion reactions observed at higher concentrations. Daratumumab is initially administered as 16 mg/kg every 2 weeks for 3–4 doses, then given monthly with further doses spaced out pending clinical response. To further minimize infusion reactions, patients receiving daratumumab were premedicated with acetaminophen 15 mg/kg (max 1000 mg), diphenhydramine 1 mg/kg (max 50 mg), famotidine 0.5 mg/kg (max 20 mg), montelukast 4–10 mg, and methylprednisolone 1.5 mg/kg (max 100 mg) prior to the first dose and 1 mg/kg (max 60 mg) prior to subsequent doses. Patients also received post‐medication with prednisone 1 mg/kg (max 60 mg) for 24–48 h following the daratumumab infusion.

## 2 Case Descriptions

### 2.1 Patient 1

**FIGURE 1:**
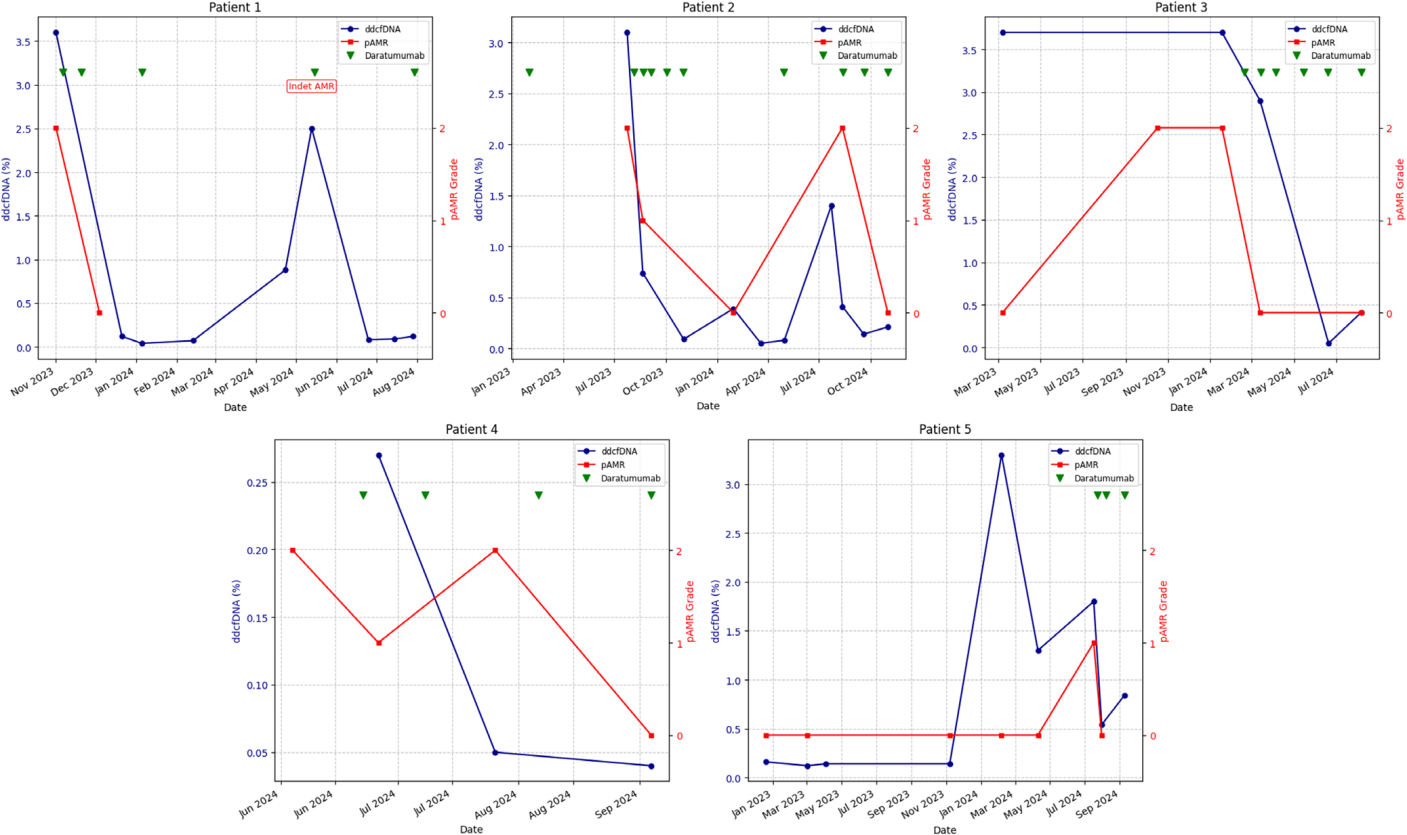
Clinical course of Patients 1–5. dd‐cfDNA, donor‐derived cell‐free DNA; Indet AMR, indeterminate pAMR; pAMR, pathologic antibody‐mediated rejection.

**FIGURE 2:**
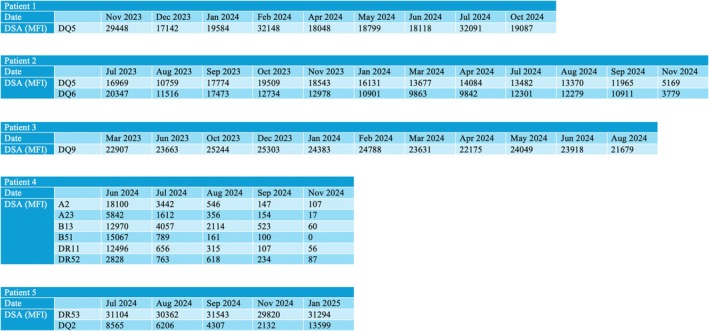
Donor specific antibodies (DSA) levels in Patients 1–5. MFI, mean fluorescence intensity.

Three‐year‐old male with a history of tricuspid atresia with pulmonary atresia and left ventricle non‐compaction cardiomyopathy who underwent HT at 7 months of age. He was maintained on tacrolimus and sirolimus for immunosuppression. He was found to have de novo donor‐specific antibody (dnDSA) (HLA‐DQ5 = 29 448 mean fluorescence intensity [MFI]) and high dd‐cfDNA fraction (Figure 1) in the blood. His EMB revealed findings consistent with pAMR2. He underwent treatment with IVIG, rituximab, and daratumumab. A repeat endomyocardial biopsy showed complete resolution of AMR. The dd‐cfDNA levels normalized as well. His DSA MFI levels declined following treatment but remained persistently elevated (Figure 2). It is important to note that although DSA MFI values were consistently elevated, there was a fourfold reduction in titer when comparing DSA levels between AMR+ and AMR− (Table 1). He received two more doses of IVIG and daratumumab, and his therapies were discontinued. However, 4.5 months later, he had an increased dd‐cfDNA fraction on routine surveillance. His EMB was indeterminate for AMR. He subsequently was treated again with IVIG and daratumumab. His dd‐cfDNA fraction decreased again and remains low with ongoing periodic treatments.

**TABLE 1:** Biopsy and corresponding DSA testing results after administration of rejection treatment.

	Age (years)	Initial biopsy pAMR grade	Days until AMR− (pAMR = 0)	AMR number of DSAs	AMR+ (highest DSA titer)	AMR− (highest DSA titer)	HLA CI DSA (AMR−)	HLA CII DSA (AMR−)	Titer reduction (fold)
Patient 1	3	2	33	1	1:4096	1:1024	None	DQ5	4
Patient 2	15	2	71	2	1:256	1:64	None	DQ5 DQ6	4
Patient 3	15	2	148	1	1:256	1:128	None	DQ9	2
Patient 4	12	2	92	7	1:64	No DSA	None	None	64
Patient 5	18	1	14	2	1:1024	1:1024	None	DR53 DQ2	0

### 2.2 Patient 2

Fifteen‐year‐old male with a history of dilated cardiomyopathy who underwent HT at 14 years of age. He was found to have acute cellular rejection and AMR (3A/2R, pAMR2) on routine surveillance 2 years post‐HT. His blood work also showed dnDSA (DQ5 = 17 000 MFI, DQ6 = 20 300 MFI) and high dd‐cfDNA fraction (Figure 1). He was treated with multiple rounds of plasmapheresis, IVIG, rituximab, and daratumumab. The dd‐cfDNA levels normalized, and his follow‐up biopsy was negative for rejection after three doses of daratumumab. On routine surveillance after discontinuing treatment for AMR, he was again noted to have high dd‐cfDNA levels, which prompted a repeat biopsy that showed pAMR2. Treatment with daratumumab was restarted, and his dd‐cfDNA fraction has once again decreased. His most recent biopsy now shows pAMR 0, and he remains on periodic daratumumab treatments with ongoing close monitoring. DSA levels are currently declining with treatment (Figure 2).

### 2.3 Patient 3

Fifteen‐year‐old female with a history of hypertrophic cardiomyopathy who underwent HT at 13 years of age. Two years post‐HT she was noted to have elevated DSA (DQ9 = 30 651 MFI), high dd‐cfDNA levels, and her biopsy showed AMR and acute cellular rejection (pAMR2, 2/1R). Before our center's use of daratumumab for AMR, she was treated with plasmapheresis, IVIG, bortezomib, and rituximab. She was again noted to have pAMR2, 3 months after therapy. She again underwent treatment with IVIG and rituximab. Despite regular IVIG and rituximab, she had persistent pAMR and grossly elevated dd‐cfDNA. Thus, she was transitioned to IVIG and daratumumab therapy, and follow‐up biopsy showed pAMR0 and her dd‐cfDNA decreased sharply with treatment (Figure 1). Her DSA MFI remains persistently elevated; however, like Patient 1, she observed a twofold reduction in titer when comparing DSA levels between AMR+ and AMR− (Figure 2 and Table 1).

### 2.4 Patient 4

Twelve‐year‐old male with a history of hypoplastic left heart syndrome status post Fontan palliation with Fontan circulatory failure who underwent HT at 12 years of age. He was found to have elevated DSA (A2 = 18 100 MFI, A23 = 5842 MFI, B13 = 12 970 MFI, B51 = 15 067 MFI, DR11 = 12 496 MFI, DR52 = 2828 MFI) 1 week post‐HT and biopsy showed pAMR2. His dd‐cfDNA fraction was also elevated. He was treated with plasmapheresis, IVIG, rituximab, and daratumumab. DSA and dd‐cfDNA levels improved and repeat biopsy after 8 weeks was negative (Figures 1 and 2). Treatment intervals are gradually being spaced out over longer intervals.

### 2.5 Patient 5

Eighteen‐year‐old female with a history of left ventricular non‐compaction who underwent HT at 9 years of age. Her transplant was complicated by recurrent rejection and coronary vasculopathy. Thus, she underwent a repeat HT at 17 years of age. She was found to have both cellular and AMR (1B/1R, pAMR 1) on routine biopsy 2 years post‐HT. She was noted to have dnDSA (DR53 = 31 104 MFI, DQ2 = 8565 MFI) and elevated dd‐cfDNA fraction. She received IVIG, rituximab, and daratumumab. Her pAMR and dd‐cfDNA improved (Figure 1) with consistently elevated DSA levels as observed by MFI after treatment (Figure 2).

## 3 Discussion

This case series highlights the successful use of daratumumab, an anti‐CD38 monoclonal antibody, in the treatment of AMR in pediatric and young adult HT recipients. Daratumumab, an anti‐CD38 monoclonal antibody, targets plasma cells responsible for producing pathogenic donor‐specific antibodies (DSA) in AMR, making it an innovative treatment option for refractory AMR, particularly in cases where conventional therapies have failed. This has been especially evident in kidney transplant recipients [7, 9]. In our case series, daratumumab was successfully used alongside standard treatments (IVIG, rituximab, plasmapheresis), leading to reductions in dd‐cfDNA and improvements in biopsy findings. These results are consistent with other reports which highlighted daratumumab's effectiveness in refractory AMR, demonstrating its ability to deplete CD38+ plasma cells, reduce dd‐cfDNA levels, and achieve histologic improvement, all while maintaining a favorable safety profile [5, 10]. Furthermore, these findings support the understanding that rejection is a multifactorial process involving various immune cell populations. The observed impact of daratumumab on rejection in this cohort suggests that its efficacy may extend beyond just plasma cell depletion, likely influencing other immune cell subsets involved in certain types of rejection [11, 12]. Daratumumab targets not only plasma cells responsible for producing antibodies in AMR, but also depletes CD38‐expressing natural killer (NK) cells, which are implicated in the pathogenesis of AMR through mechanisms such as antibody‐dependent cellular cytotoxicity and microvascular inflammation. This dual mechanism is particularly relevant in refractory AMR. Emerging evidence from case series and translational studies supports this approach, demonstrating that targeting both humoral and cellular components of AMR can lead to clinical and molecular remission, even in refractory cases [11, 13, 14].

The cases presented in this series also highlight the utility of dd‐cfDNA as a dynamic biomarker for monitoring AMR activity. In our patients, the observed association between rising dd‐cfDNA levels and AMR episodes underscores its potential value as a biomarker when treating for AMR. Notably, treatment with daratumumab in combination with other agents led to a reduction in dd‐cfDNA levels, reflecting therapeutic efficacy and resolution of AMR in biopsy samples. This trend of dd‐cfDNA levels paralleling histologic findings supports previous studies that suggest dd‐cfDNA not only detects rejection but also correlates with therapeutic outcomes. In line with other studies, the detection of elevated dd‐cfDNA in these patients was shown to precede EMB diagnosis, allowing for earlier diagnosis and initiation of therapy [15, 16]. Our case series supports this finding, highlighting the ability of dd‐cfDNA to guide clinical decision‐making by detecting AMR before it becomes clinically apparent.

However, as evidenced by Patients 3 and 5, even when dd‐cfDNA levels normalized and AMR resolved with therapy, DSA (especially class II) continued to persist when monitoring by MFI values alone. While Patients 2 and 4 showed a decrease in DSA MFI levels, it is worth noting that MFI values might not be the most precise indicator for detection of HLA antibodies. Assessment of HLA antibody titers through dilution studies has been shown to better reflect the true presence of HLA antibody [17]. In four out of the five cases, titers calculated for the highest‐ranking DSA were reduced by a median of fourfold when comparing DSA levels between AMR+ and AMR− time points (Table 1). Despite the reduction in titer, most of the patients had persistent DSA suggesting variable antibody responses despite clinical improvement. This variability may reflect daratumumab's actions through different mechanisms, not only depleting plasma cells and reducing antibody production, but also modulating other immune pathways involved in AMR. Furthermore, HLA protein expression is dynamic, demonstrating differential expressions between loci and tissue types [18]. Studies have shown that HLA‐DQ exhibits lower expression compared to other classical CII HLA loci such as HLA‐DR [18]. The degree of expression exhibited by HLA‐DQ may explain the lack of pAMR in the setting of persistent Class II antibodies, although their potential impact on long‐term outcomes remains a concern.

Taken together, these findings raise important questions about the clinical significance of DSA MFI values and whether they should be relied upon as the sole indicator for guiding treatment decisions. Notably, some patients demonstrated clinical and molecular improvement, such as resolution of AMR and normalization of dd‐cfDNA despite persistently elevated DSA MFI levels. This highlights the potential limitations of MFI as a standalone marker and emphasizes the need to interpret DSA results within the broader clinical context.

We also acknowledge the concern regarding the recurrence of AMR following the discontinuation of therapy. Persistent or de novo DSAs have been associated with an increased risk of chronic rejection and long‐term allograft loss, reinforcing the need for ongoing surveillance [19, 20]. These risks underscore the importance of individualized management strategies that incorporate not only DSA measurements but also functional markers such as dd‐cfDNA and graft histology to more accurately assess immune activity and guide clinical action. In this context, dd‐cfDNA emerges as a valuable tool for monitoring treatment response, particularly in patients with persistently elevated DSA MFI values who remain clinically stable.

Despite the promising results, there are several limitations. First, while daratumumab has been shown to be effective in treating AMR, we still lack consensus on the optimal treatment duration and frequency for daratumumab therapy. In our case series, treatment strategies varied case by case, from more frequent infusions initially, which were then gradually spaced out as dd‐cfDNA levels normalized along with clinical and pathological improvement. Moreover, while dd‐cfDNA is a promising non‐invasive biomarker of graft injury, at this time it cannot fully replace EMB in identifying graft pathology. Finally, the small number of patients and observational nature of this series limit the generalizability of the findings. We hope this series will spur future work to confirm our observations and better inform the use of dd‐cfDNA with certain treatments (e.g., daratumumab) in AMR.

## 4 Conclusion

In conclusion, this case series contributes to the growing evidence that dd‐cfDNA may serve as a valuable biomarker in the diagnosis and management of AMR post‐HT. The addition of daratumumab to the therapeutic armamentarium offers a promising approach to treating AMR, particularly when conventional therapies fail. Future studies are necessary for confirming the efficacy of daratumumab and to guide optimal treatment strategies, with dd‐cfDNA serving as a surrogate endpoint for assessing therapeutic response.

## Conflicts of Interest

The authors declare no conflicts of interest.

## Data Availability

The authors have nothing to report.
